# Evidence for genetic variation in Natterer’s bats (*Myotis nattereri*) across three regions in Germany but no evidence for co-variation with their associated astroviruses

**DOI:** 10.1186/s12862-016-0856-0

**Published:** 2017-01-05

**Authors:** Tanja K. Halczok, Kerstin Fischer, Robert Gierke, Veronika Zeus, Frauke Meier, Christoph Treß, Anne Balkema-Buschmann, Sébastien J. Puechmaille, Gerald Kerth

**Affiliations:** 1 Ernst-Moritz-Arndt Universität Greifswald, Zoological Institute and Museum, Soldmannstr. 14, 17489 Greifswald, Germany; 2 Friedrich-Loeffler-Institut, Institute of Novel and Emerging Infectious Diseases, Suedufer 10, 17493 Greifswald, Insel Riems Germany; 3 Echolot GbR, Eulerstr. 12, 48155 Münster, Germany; 4 Fledermausforschungsprojekt Wooster Teerofen e.V., Gartenstraße 4, 98617 Meiningen, Germany

**Keywords:** Genetic, Population structure, *Myotis nattereri*, Astrovirus, Host, Gene flow

## Abstract

**Background:**

As bats have recently been described to harbor many different viruses, several studies have investigated the genetic co-variation between viruses and different bat species. However, little is known about the genetic co-variation of viruses and different populations of the same bat species, although such information is needed for an understanding of virus transmission dynamics within a given host species. We hypothesized that if virus transmission between host populations depends on events linked to gene flow in the bats, genetic co-variation should exist between host populations and astroviruses.

**Results:**

We used 19 nuclear and one mitochondrial microsatellite loci to analyze the genetic population structure of the Natterer’s bat (*Myotis nattereri*) within and among populations at different geographical scales in Germany. Further, we correlated the observed bat population structure to that of partial astrovirus sequences (323–394 nt fragments of the RNA-dependent RNA polymerase gene) obtained from the same bat populations. Our analyses revealed that the studied bat colonies can be grouped into three distinct genetic clusters, corresponding to the three geographic regions sampled. Furthermore, we observed an overall isolation-by-distance pattern, while no significant pattern was observed within a geographic region. Moreover, we found no correlation between the genetic distances among the bat populations and the astrovirus sequences they harbored. Even though high genetic similarity of some of the astrovirus haplotypes found in several different regions was detected, identical astrovirus haplotypes were not shared between different sampled regions.

**Conclusions:**

The genetic population structure of the bat host suggests that mating sites where several local breeding colonies meet act as stepping-stones for gene flow. Identical astrovirus haplotypes were not shared between different sampled regions suggesting that astroviruses are mostly transmitted among host colonies at the local scale. Nevertheless, high genetic similarity of some of the astrovirus haplotypes found in several different regions implies that occasional transmission across regions with subsequent mutations of the virus haplotypes does occur.

**Electronic supplementary material:**

The online version of this article (doi:10.1186/s12862-016-0856-0) contains supplementary material, which is available to authorized users.

## Background

With their particular social, ecological, physiological and immunological traits, bats provide unique hosts for many viruses to co-evolve with (e.g. [1, 2]). Indeed, bats are increasingly recognized as reservoirs for a wide range of viruses, some of which carry a zoonotic potential, for example rabies and other viruses of the genus Lyssavirus, SARS-like, MERS-like and other coronaviruses [2–5]. Consequently, several studies have investigated the genetic co-variation between different bat species and their associated viruses [6, 7]. However, much less is known about genetic co-variation of viruses and different populations of the same bat species, although such information is required to gain a better understanding of the transmission dynamics within a given host species (e.g. [8, 9]).

The Natterer’s bat (*Myotis nattereri* Kuhl 1817 *sensu lato* [10]) is a non-migratory vespertilionid bat that is widespread throughout Europe with the exception of the Iberian and Italian peninsula and the South of France [11]. While this species uses underground sites for hibernation during the winter months, it mostly roosts in trees and buildings during summer forming maternity colonies that consist of female bats and their juveniles as well as occasionally some males [12, 13]. Males typically roost either individually or in small groups in the vicinity of the maternity colonies [14]. Male and female Natterer’s bats have been found to exhibit philopatry even though males leave their natal colony but stay in its vicinity [13]. Mating takes place during autumn at swarming sites that are typically up to 50 km away from the summer colony [13].

Recently, various viruses have been reported to be harbored by *M. nattereri* [15], including herpes- [16], lyssa- [17] and astroviruses (e.g. [18]). The *Astroviridae* form a large family of non-enveloped, positive-sense, single-stranded RNA viruses [19]. Astroviruses are mostly transmitted via the fecal-oral route [20] and may cause diarrhea in many animal species, including humans [21]. However, the route of transmission in bats has not yet been elucidated. Even though astroviruses have been detected in a variety of species [22], bats have been hypothesized to be a potential reservoir host in Europe and Asia [7, 23]. The high prevalence and diversity of astroviruses harbored by bats is remarkable [18, 21, 23] and their capability to cross species barriers and become adapted to new hosts, including spill-over to other taxa, has been suggested [24]. Due to the occurrence of astroviruses in animals in close contact to humans, e.g. livestock and also bats using human habitation as roosting, it has been argued that astroviruses should be considered as potential candidates for zoonotic infections (e.g. [24]). However, almost nothing is known about the transmission of astrovirus among different populations of their bat hosts.

We studied patterns of population genetic structure and dispersal of *Myotis nattereri* within and among three geographic regions of Germany using both nuclear and mitochondrial microsatellite markers. The population genetic structure of *M. nattereri* has previously only been investigated in the United Kingdom (UK; [13]). However, the population genetic structure of bats occurring in the UK may be affected by their insular status, and some important differences between insular and continental populations have been described (e.g. [25]). Thus, our study adds new important insights into the dispersal behavior of *M. nattereri* in mainland Europe*.* Moreover, we investigated for the first time if genetic co-variation occurs between populations of a bat host and its harboured astroviruses. Fischer et al. [18] reported distinctly higher similarities in astrovirus sequences of samples collected from the same bat species in different geographic localities than between samples from different species sampled at the same locality, whereas different results were obtained in the Czech Republic for some other European bat species [26]. Because our analyses are based on the sequences found by Fischer et al. [18] in *M. nattereri*, we assume that, similarly to coronaviruses in Chinese bats [27], astroviruses are mostly transmitted within Natterer’s bats rather than within the local bat community as a whole. We hypothesized that if virus transmission between host populations were associated with events linked to gene flow in the bats, e.g. mating [28], genetic co-variation should be detectable between host populations and astroviruses on a larger scale (e.g. between geographic regions), even though not necessarily within a certain region.

Many bat astroviruses form distinct phylogenetic clusters [21], but little is known whether astrovirus population structure matches that of their host species. As astroviruses are hypothesized to be transmitted via the fecal-oral route [20], both direct and indirect virus transmission within the breeding colonies of bats and at swarming sites during mating seem possible [8]. In comparison, for bat ectoparasites such as bat flies (*Nycteribiidae*) that are also transmitted both by direct body contact and indirectly through the bats’ roosts, a more efficient transmission among different bat populations has been detected at swarming sites as compared to breeding colonies [29]. As swarming sites represent the main mating sites for *M. nattereri,* where gene flow occurs, it is expected that if viruses are also mainly transmitted between conspecifics during that period, the transmission route of the viruses should resemble the pattern of host gene flow. Since transmission of astroviruses at swarming sites would cause viruses to be transmitted between members of different colonies visiting a given swarming site, local differentiation should not occur at the colony level but rather on a larger scale. Viral genetic patterns should therefore follow an isolation-by-distance pattern using swarming sites as stepping-stones for gene flow, as suggested for the bat host [30].

## Methods

### Study area and sample collection

**Fig. 1:**
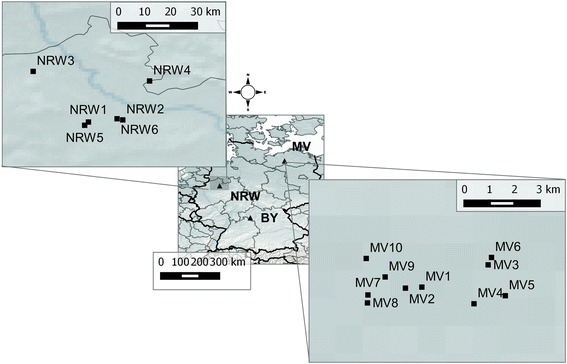
Outline of the study area and sampling localities of *Myotis nattereri* samples in Germany. The *triangular* markings represent the sampling areas within Bavaria (BY), Mecklenburg Western Pomerania (MV) and North Rhine Westphalia (NRW). The close-up maps for NRW on the upper left and for MV on the lower right show the exact sampling localities of the colonies. As in BY only one colony was sampled, no close-up is provided

Sampling occurred between 2010 and 2014 from maternity colonies in three regions in Germany: Bavaria (BY, *N* = 92 adult females, one colony), North Rhine Westphalia (NRW, *N* = 100 adult females, five colonies) and Mecklenburg Western Pomerania (MV, *N* = 172 adult females, ten colonies; Fig. 1). Bats were either taken directly out of the bat boxes provided for the colonies or captured using mist nets or harp traps when leaving the colonies’ roosts. Three millimeter wing tissue samples were collected and stored in 90% ethanol until DNA extraction. In addition, between 2012 and 2014, samples of bat saliva, feces and urine were opportunistically taken within the three regions of interest (BY, *N* = 177; NRW, *N* = 74; MV, *N* = 19) and screened for the presence of astrovirus-related RNA (Additional file 1: Table S3).

### Bat DNA extraction and amplification

Genomic DNA was extracted using an ammonium acetate precipitation method [31]. Individuals were genotyped using 19 nuclear microsatellite markers and one mitochondrial microsatellite marker. The following nuclear markers were used: EF15 [32]; b22 [33]; A2-Mluc, A13-Mluc, E5-Mluc, G6-Mluc, G30-Mluc, G31-Mluc [34], D15, H19, H23, H29 [35]; Mnatt-1, Mnatt-2 [36]; Mschreib3 [37]; Mnatt-8, Mnatt-11 [38]; FV5AP [39] and GZBYR (5′-TCCTTGTCACTATAAGCTCAGTGG-3′ (forward); 5′-CCAGGCAATAGTCTCCTAGCAC-3′ (reverse)). The 5′ end of the reverse primers FV5AP and G30-Mluc were PIG-tailed [40] with the sequence GTTT and the 5′ end of the reverse primer G31-Mluc with the sequence GTTTT to facilitate adenylation. These 19 autosomal microsatellite markers were amplified in two multiplex polymerase chain reactions (PCRs, Table 1). PCRs were carried out in 9 μl reaction volumes using the Qiagen multiplex Kit (Qiagen, Hilden, Germany). Each multiplex reaction contained 1 x Qiagen Multiplex Master Mix and between 0.11 μM and 1.06 μM of each primer. After drying 1 μl of DNA (approximately 10 ng) for 15 min at 52 °C in a 96-well PCR plate (VWR), multiplex reactions were performed. The PCR amplification was carried out in a 2720 Thermal Cycler (Applied Biosystems), with an initial 5 min denaturation at 95 °C, followed by 30 cycles with denaturation at 95 °C for 30 s, annealing at 60 °C for 90 s and extension at 72 °C for 1 min. Final incubation occurred at 60 °C for 30 min.

**Table 1:** Genetic diversity at nuclear loci within *Myotis nattereri* populations sampled within Germany

	*Final concentration* *[μM]*	*BY*					*NRW*					*MV*				
		*N*	*A*	*H*_*e*_	*H*_*o*_	*Ar*	*N*	*A*	*H*_*e*_	*H*_*o*_	*Ar*	*N*	*A*	*H*_*e*_	*H*_*o*_	*Ar*
*Multiplex 1*																
*A2-Mluc*	0.22	92	11	0.78	0.76	10.93	100	10	0.63	0.63	9.72	168	13	0.82	0.82	12.50
*b22*	0.22	92	7	0.75	0.78	6.94	100	8	0.78	0.74	7.70	172	8	0.79	0.77	6.99
*D15*	0.22	92	10	0.87	0.92	10.00	100	10	0.86	0.86	10.00	172	11	0.80	0.85	10.45
*EF15*	0.44	92	4	0.51	0.41	4.00	100	4	0.59	0.51	3.86	172	5	0.64	0.62	4.88
*G30-Mluc*	0.22	92	19	0.88	0.90	18.86	97	20	0.83	0.80	19.41	170	22	0.83	0.78	19.10
*G6-Mluc*	0.22	92	8	0.82	0.78	7.94	100	9	0.82	0.85	8.96	172	9	0.84	0.83	8.75
*H23*	0.22	92	9	0.80	0.83	8.99	100	11	0.82	0.81	10.86	172	11	0.79	0.80	10.49
*H29*	0.56	92	6	0.69	0.71	6.00	100	8	0.72	0.67	7.84	172	8	0.68	0.63	6.63
*Mnatt-1*	0.11	91	7	0.70	0.74	6.94	100	5	0.64	0.69	5.00	172	7	0.69	0.68	6.63
Mnatt-11*	0.56	91	8	0.83	0.85	7.94	100	9	0.81	0.78	8.70	172	10	0.85	0.88	9.47
*Mnatt-8*	0.56	86	19	0.90	0.88	19.00	99	25	0.93	0.88	24.56	172	26	0.93	0.85	22.31
*Multiplex 2*																
*A13-Mluc*	0.44	89	16	0.85	0.91	15.87	100	16	0.83	0.80	15.58	172	14	0.88	0.90	13.74
*E5-Mluc*	1.06	87	10	0.80	0.78	10.00	100	13	0.81	0.76	12.70	172	12	0.81	0.80	10.97
*FV5AP*	0.22	91	4	0.68	0.59	4.00	100	5	0.65	0.63	5.00	172	7	0.70	0.75	6.44
*G31-Mluc*	0.22	92	6	0.80	0.79	6.00	99	8	0.81	0.81	7.87	172	8	0.79	0.79	7.75
*GZBYR*	0.22	92	9	0.78	0.74	9.00	100	10	0.83	0.78	9.86	171	10	0.83	0.81	9.86
*H19*	0.33	92	18	0.87	0.83	17.93	100	15	0.89	0.88	14.70	171	17	0.83	0.78	15.90
Mnatt-2*	0.22	92	8	0.83	0.85	8.000	100	9	0.81	0.78	8.70	170	9	0.85	0.86	8.97
*Mschreib3*	0.22	87	16	0.90	0.84	15.99	99	17	0.90	0.85	16.60	169	21	0.93	0.86	20.08

*N* number of samples successfully analyzed (total number of individuals: Bavaria (BY) 92, North Rhine Westphalia (NRW) 100, Mecklenburg Western Pomerania (MV) 172); *A* number of alleles; *H*_*o*_ observed heterozygosity; *H*_*e*_ expected heterozygosity; (A_R_) allelic richness based on a minimum of 74 individuals. The primers marked with an asterisk (*) appear to be linked. Forward markers were dyed as follows: *6-FAM*: A13-Mluc, D15, b22, G30-Mluc, H19, H29, GZBYR, FV5AP, Mnatt-1, Mnatt-2; *VIC*: Mnatt-8, G31-Mluc, H23; NED: A2-Mluc, EF15, Mschreib3, E5-Mluc and *PET*: Mnatt-11, G6-Mluc

The mitochondrial DNA marker AT-2 [41] was amplified in a separate PCR. After drying 1 μl of DNA (approximately 10 ng) for 15 min at 52 °C in a 96-well PCR plate (VWR), this PCR was carried out in 10 μl reaction volume which contained 0.2 μM of Primer AT-2, 1.0 mM dNTPs, 0.8 mM of MgCl_2_, 1.0 μl 10 x *Taq* Buffer B1 (Solis, BioDyne, Tartu, Estonia) and 1 unit of *Taq* Hot FirePol® DNA Polymerase (Solis, BioDyne, Tartu, Estonia). This PCR amplification was carried out in a 2720 Thermal Cycler (Applied Biosystems), with an initial 15 min denaturation at 95 °C, followed by 40 cycles with denaturation at 95 °C for 30 s, annealing at 53 °C for 60 s and extension at 72 °C for 1 min. Final incubation occurred at 72 °C for 7 min.

PCR products were separated using an ABI 3130 Genetic Analyzer (Applied Biosystems) together with the internal size standard Genescan 500 liz (Applied Biosystems) and analyzed using Genemapper v 5.0 (Applied Biosystems).

### Data analysis

#### Bat population genetic structure

Since it has been reported that the presence of closely related individuals within populations can bias Bayesian multi-locus clustering methods [42], we removed closely related individuals from the dataset before conducting population genetic structure analyses using the program Structure [43]. For this purpose, we first determined the degree of relatedness between all pairs of individuals within a population using TrioML [44], as implemented in the Coancestry 1.0.1.5 software package [45]. Relatedness densities were further plotted using R (R Core [46]) in order to determine the relatedness threshold for excluding individuals from the analyses. This threshold was selected manually by best separating the first peak of the plotted distribution (i.e. closely related individuals) from the rest (unrelated individuals). From every pair of individuals with a relatedness value exceeding the determined threshold (0.3), one individual was randomly removed, respectively. All other analyses, except for Structure, were performed on the whole data set. As uneven sampling can bias inferences on the number of clusters in the program Structure [47], efforts were made to have comparable number of individuals from the three regions investigated after the removal of closely related individuals (71 from BY, 73 from NRW and 104 from MV; cf. Results).

As preliminary runs using the original Structure model showed limited population structure (Additional file 1: Figure S2), Structure [43] was run on the nuclear DNA dataset assuming admixture and correlated allele frequencies using the LOCPRIOR model that allows for the use of sample group information (here the colony) in the clustering process [48]. Thus, twenty independent runs of *K* = 1–10 were conducted for the whole dataset after removing closely related individuals as mentioned above. Additionally, twenty independent runs of *K* = 1–10 were run for each dataset within a sampling region (NRW, MV and BY), respectively. All runs used 10^6^ iterations after a burn-in period of 10^5^.

For each of the genetically distinct populations inferred by Structure the significance of deviations from Hardy-Weinberg equilibrium (HWG, [49]) and linkage disequilibrium between loci was tested in Genepop 4.0.7 [50]. The False Discovery Rate (FDR) correction method was used to deal with multiple testing [51].

To assess the level of genetic diversity, the observed (*H*_*o*_) and expected heterozygosity (*H*_*ex*_) for each locus as well as for each population inferred by Structure for the complete data set were calculated using Genetix 4.05.2 [52]. The mean number of alleles (*A*) and the allelic richness (*A*_*R*_) were calculated for each locus and each subpopulation using Fstat v.2.9.3 [53] (Table 1). Differences in the number of alleles per locus, allelic richness and expected and observed heterozygosities between the populations inferred by Structure were tested for significance using the Wilcoxon signed-rank test in R. MICRO-CHECKER 2.2.3 [54], set for 10 000 iterations and a 95% confidence interval, was used to test for null alleles.

Population pairwise *F*_*ST*_ values [55] on the whole dataset including closely related individuals, were used to measure the level of genetic differentiation between the populations inferred by Structure. For both the nuclear and the mitochondrial DNA data set, a hierarchical population structure was assumed where colonies were clustered within regions. *F*_*ST*_ values were thus calculated using hierarchical analyses within the R-package HierFstat [56, 57]. Genetic structure was tested among colonies within sampling regions with more than 1 colony (MV and NRW) and among sampling regions. The significance of the *F*-statistics was tested by 10,000 permutations.

Isolation-by-distance for the entire set of 17 colonies was tested via Mantel’s test [58] from the comparison of all pairwise *F*_ST_/(1-*F*_ST_) values with pairwise geographic distances using the R package ecodist [59] with 10,000 permutations. The test was performed for the whole dataset as well as within regions consisting of several colonies (NRW and MV).

#### Correlation of bat and virus genetic distances

A total of 270 samples obtained from saliva, feces and urine of *Myotis nattereri* were screened for the presence of astroviruses by using a published hemi-nested PCR protocol [21] for the amplification of a highly conserved region of the RNA dependent RNA polymerase gene (RdRp). This PCR assay enables the detection of a variety of bat-associated astroviruses by using degenerated primers. Further details of the virus-related sampling and laboratory are presented in Fischer et al. [18]. A total 57 sequences representing 19 different astrovirus haplotypes (=sequences having 100% identity) (N1-19, Fig. 4) were identified from the screening of 270 samples. Three haplotypes were excluded from the analyses (N17-19, Fig. 4) as they were phylogenetically too distant from the remaining 16 and too rarely encountered (only once per haplotype) to make biologically meaningful inferences about their correlation to the bat host’s population genetic structure. Out of the 270 samples, Fischer et al. [18] were able to assign 73 individual sequences to a specific haplotype (N1-19, Fig. 4), whereas the remaining astrovirus positive samples were shorter than 279 nt and could therefore not be assigned to a haplotype. As in this study we only analyzed individually marked adult female bats that were clearly identifiable via their RFID-tag, in total 57 individual sequences, representing these 16 different haplotypes, were used (N1-16, Fig. 4).

The genetic distances between the different astrovirus haplotypes were calculated using the Tamura-Nei model implemented in Mega 6.0 [60]. Further, the sequences were translated into amino acids and amino acid genetic distances were computed using the *p*-dist method implemented in Mega 6.0 [60]. This latter measure was tentatively used to differentiate viral species following the recommendation of the *Astroviridae* working group of the International Committee for Taxonomy of Viruses (ICTV Astroviridae Working [61]), proposing that mean amino acid genetic distances (*p*-dist) of the full length ORF2 larger than 0.284 distinguish different species.

As we found no shared identical sequences between regions (BY, MV and NRW), a permutation test was conducted using R (R Core [46]) to test how likely such a pattern would be expected by chance (Additional file 1: Database S1).

Moreover, the program Poptree2 [62] was used to generate genetic distance matrices for the nuclear and mitochondrial DNA datasets based on the D_A_ distances [63] of population microsatellite allele frequencies within a priori populations. For the astrovirus dataset, we first used the program jModeltest 2.1.7 [64] to calculate likelihood scores for substitution model selection. Genetic and amino acid distances were then calculated using Mega 6.0 [60] based on the Maximum Composite Likelihood substitution model with gamma correction for among-site rate heterogeneity and an estimated proportion of invariable sites.

Associations between astrovirus and bat host genetic distances (both mitochondrial and nuclear) were first analyzed via a Mantel Test [58] using the software GenAlex 6.501 [65]. To control for the possible effect of geographic distance, we also carried out partial Mantel tests in PASSaGE v 2.0.11.6 (9999 permutations, [66]). The geographic distance matrix used was calculated from the GPS coordinates of the different sampling sites using the Create option in PASSaGE.

For the genetic correlations between host and astrovirus sequences the NRW dataset was modified. Due to significant population genetic structuring on the basis of mtDNA and the fact that the sampled colonies in NRW are up to 46 km apart from each other compared to maximally 6.5 km in MV (see Fig. 1) we split the NRW data set in four separate sampling units (NRW2, NRW3, NRW5 and NRW6; Fig. 1). Here, we only used genetic data from the colonies within NRW where virus sequences were detected. Together with MV and BY, the total dataset for comparing host and virus population structures now consisted of six populations between which pairwise genetic distances were computed as mentioned above.

## Results

### Bat population genetic structure

**Fig. 2:**
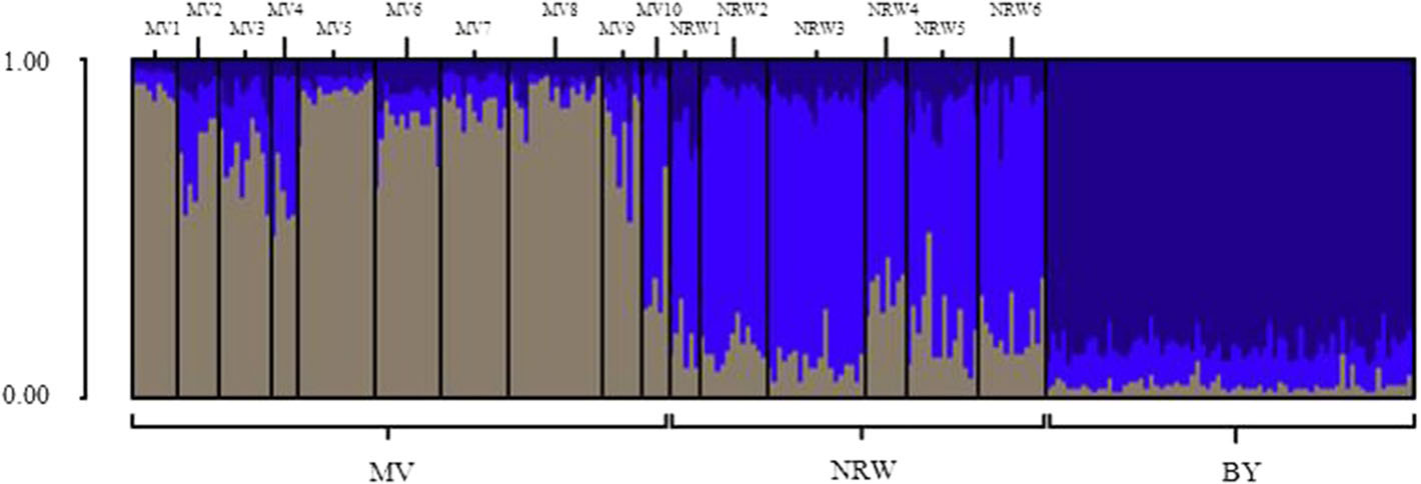
Bar plot graph of estimated membership coefficient of *Myotis nattereri* from Bayesian analysis for *K* = 3 generated using Structure and the Locprior option. For the summary of the log-likelihood values from the 20 independent runs conducted with Structure see Additional file 1: Figure S1

After removing closely related individuals at the threshold of 0.3, the nuclear DNA dataset consisted of samples from 248 bats in total, including 71 from BY, 73 from NRW and 104 from MV. Using the 19 autosomal microsatellite loci, Structure inferred the presence of three distinct genetic clusters (Fig. 2; Additional file 1: Figure S1), splitting our data set into the three sampled regions NRW, MV and BY. No additional sub-structuring was detected by Structure within any of these three sampling regions (data not shown).

The 19 autosomal microsatellite loci had between 4 and 24 alleles and an average of 10.2–11.7 alleles per each of the three genetically distinct populations inferred by the program Structure (Table 1). No significant deviations from Hardy–Weinberg equilibrium were detected in these three populations. Deviations from linkage disequilibrium at the α = 0.05 level (after FDR correction) did occur consistently in all three regions between two loci (Mnatt-2 and Mnatt-11, Table 1) but were not detected in all respective colonies (linkage occurred in BY, in three out of ten colonies in MV and in three out of five colonies in NRW). Because of the inconsistencies at the colony level, we decided to nevertheless keep both loci for further analyses. No marker with consistently appearing null alleles was found within NRW and MV, whereas in BY the two loci EF15 and FV5AP showed the presence of null alleles. However, since the estimated frequency of null alleles per locus was low (<0.1), we kept those loci.

Mean expected and observed heterozygosity were globally similar across colonies and regions (Tables 1 and 2). The number of alleles found in MV was significantly higher than in BY (*P* = 0.0020) and NRW (*P* = 0.0284), but BY and NRW did not differ significantly from each other. Moreover, allelic richness was significantly higher in MV as compared to BY (*P* = 0.0124) but no significant differences were found between BY and NRW and between NRW and MV. The overall level of differentiation among the three regions was weak but significant (*F*_ST_ = 0.0088, *P* = 0.0001) based on nuclear DNA estimated using hierarchical F-statistics. Significant genetic differentiation was further identified among colonies within regions (*F*_ST_ = 0.0194, *P* = 0.0001). When analyzing the data obtained by the mitochondrial DNA marker AT-2, pairwise genetic differentiation was found to be much higher at both geographic scales (among regions: *F*_ST_ = 0.4979, *P* = 0.0001; among colonies within regions: *F*_ST_ = 0.3657, *P* = 0.0001).

**Table 2:** *P*-values for differences in number of alleles, allelic richness and observed and expected heterozygosities

	*A*	*Ar*	*Ho*	*He*
MV-NRW	0.0284*	0.6794	0.2253	0.3736
MV-BY	0.0020*	0.0124*	0.9843	0.1712
NRW-BY	0.0536	0.0894	0.1819	0.9843

*significant *p*-values at the 0.05 level

*P*-values for differences between MV, NRW and BY in number of alleles (*A*), allelic richness (*Ar*), observed heterozygosity (*H*_*o*_) and expected heterozygosity (*H*_*e*_) obtained using the Wilcoxon signed-rank test in R

**Fig. 3:**
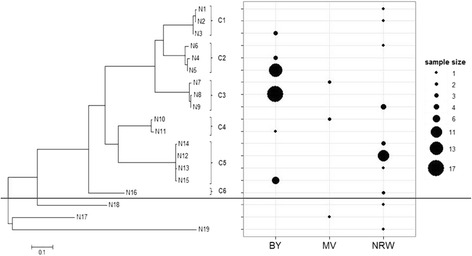
Isolation-by-distance analysis for data from 19 nuclear microsatellite loci from *Myotis nattereri* within Germany. The graph displays the significant correlation between genetic differentiation and ln of geographical distance (in km) for all pairwise comparisons of colonies. Genetic distance was measured as Rousset’s *F*_ST_/(1-*F*_ST_) and the relationship with geographic distance was tested using a Mantel test with 1000 permutations. The *line* represents a linear regression of this relationship and only serves an illustrative purpose

When correlating genetic differentiation *F*_ST_/(1-*F*_ST_) with ln of geographic distances between all colonies within our study area, a significant pattern of isolation-by-distance was detected (Mantel *r* = 0.2989, *P* = 0.0160; Fig. 3). In contrast, the Isolation-by-distance patterns within the NRW (Mantel *r* = -0.2762, *P* = 0.7869) and the MV population (Mantel *r* = 0.2275, *P* = 0.0535) were not significant, even though in MV there was a similar trend visible as in the entire data set.

### Correlation of bat and virus genetic distances

**Fig. 4:**
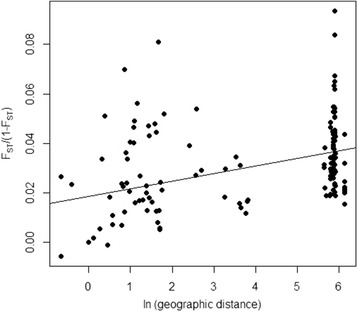
Phylogenetic tree created using the Maximum likelihood method implemented in Mega 6.0 [60] displaying the phylogenetic relationship between the 19 haplotypes of astroviruses detected in *Myotis nattereri* [18] and the occurrence of the different astrovirus haplotypes within the three regions of interest (BY = Bavaria, MV = Mecklenburg Western Pomerania, NRW = North Rhine Westphalia). C1-6 represent the putative viral species identified. The *horizontal line* separates the 16 haplotypes used for further analyses from the three remaining haplotypes (N17-19)

We found no overlap in the detected haplotypes between the three regions (Fig. 4). The permutation test revealed that the probability of having no such overlap by chance was very low (*P* = 0.007; 100,000 permutations). However, a closer look at the genetic relationship of the different astrovirus haplotypes (Fig. 4) revealed that sequences of high similarity occur in different geographic regions. Genetic distances in astrovirus haplotypes that cluster together (N1-3, N4-6, N7-9, N10-11, N12-15; Fig. 4) ranged between 0.0033 and 0.0313, whereas genetic distances among clusters were distinctly higher (0.2093–0.6895). As for the amino acid genetic distances (*p*-dist), distances ranged between 0.0221–0.0588 and 0.5732–0.6786 within and between clusters, respectively, the latter being typical of species differences. Rough estimations of divergence times using an astrovirus mutation rate of 3 × 10^−3^ [67] indicate that differences within clusters have occurred within 1–10 years, versus 70–230 years for between-cluster divergence time.

Furthermore, the astrovirus genetic distances neither correlated with those of the host’s nuclear DNA nor with those of the host’s mitochondrial DNA. This was true both for the Mantel Tests and for the partial Mantel Test correcting for geographic distance (Table 3).

**Table 3:** Mantel Test and partial Mantel Test

Substitution model	Compared dataset	*r*	*P*-value
*Mantel Test*			
	nucDNA	−0.219	0.190
	mtDNA	−0.120	0.420
*Partial Mantel Test*			
	nucDNA	−0.199	0.518
	mtDNA	−0.174	0.462

Results of the Mantel Tests and partial Mantel Tests testing for an association between astrovirus genetic distances and those of its bat host (*Myotis nattereri*), for both nuclear (nucDNA) and mitochondrial DNA (mtDNA) (*P*-value based on 1000 permutations)

## Discussion

In this study, we analyzed the population genetic structure of *M. nattereri* within and among summer colonies at different geographical scales in Germany and correlated it with that of the astrovirus sequences found in the respective host colonies. Our aim was to assess whether the population structure and dispersal patterns of host populations can explain the genetic structure of astrovirus sequences and thus ultimately shed light on the virus transmission dynamics within a given bat species [8, 9].

Our findings show significant population structure in *M. nattereri* with the detection of three genetic clusters which correspond to the three regions sampled (NRW, MV and BY). Both the results obtained using the Bayesian clustering approach and the *F*_*ST*_ values between the three genetic clusters show the existence of significant population genetic structure. The detection of an isolation-by-distance pattern over the whole study area combined with the continuous presence of the species across Germany suggests that the levels of gene flow are not high enough to prevent some population differentiation [68].

The observed strong mitochondrial substructure and weak but significant structure at the nuclear level, likely results from differences in effective population size and female philopatry combined with male-mediated gene flow. Differences in patterns of genetic structure in mitochondrial and nuclear DNA have been reported in many European bat species and were associated with male-biased dispersal [41, 69]. According to previous studies in *M. nattereri* in the UK [13, 30], both sexes appear to be highly philopatric to their natal area but visit central swarming sites during autumn for mating. According to Rivers et al. [30], this pattern results in the same genetic pattern as permanent male dispersal [13].

In connection with the existing overall population isolation-by-distance pattern detected and the absence of a significant pattern on a local scale, i.e. within a region, we suggest that individuals from different summer colonies meet and mate at swarming sites within each region (e.g. [13, 70]). This would result in gene flow following a stepping-stone model and would both lead to a significant isolation-by-distance over larger scales [71] and to the absence or a weak signal on a local scale [30, 33]. Within the UK, isolation-by-distance was not detected between summer colonies of *M. nattereri* unless distances exceeded 100 km [30]. Our results agree with those obtained by Rivers et al. [30] as we also did not detect significant genetic isolation-by-distance between summer colonies within a given region in Germany (even though there is a trend in MV), but over the study area as a whole. Therefore, we suggest that the isolation-by-distance pattern observed here is generated by swarming sites acting as stepping-stones for gene flow.

In our study area, identical astrovirus haplotypes harbored by *M. nattereri* do not overlap between geographic regions (BY, MV and NRW, Fig. 4). Based on the partial sequence of the conserved RdRp gene analysed in this study [21], where the mean amino acid distances ranged from 0.022 to 0.059 within and 0.573–0.679 between clusters, the analyzed haplotypes form six different groups which might represent six different viral species [61] (C1-6, Fig. 4). The detection of the same putative viral species in the different regions combined with the estimated divergence times (<10 years for within species) suggests occasional transmission between host populations. Both the observed genetic population structure of *M. nattereri* and existing data from ringing studies in Germany [72] show that Natterer’s bats rarely if ever move over long distances (more than 100 km). Thus, it is unlikely that individual bats directly transmit a certain virus haplotype between the three geographic regions analyzed (NRW, BY and MV). Since viruses have a considerably higher mutation rate [73] compared to the bat host, mutations in the virus sequences do occur within much shorter time scales than mutation in the bats’ genome [74, 75]. As bats need to transmit the astroviruses directly, the movement of viruses across the landscape should mirror the movement of the bats and hence occur successively over large distances following the stepping stone model of the host. As a consequence, virus transmission over large distances is likely to take multiple years. In the course of these successive transmissions events, mutations will occur in the virus which will lead to viral population differentiation as we observe within the putative viral species.

No association was found when correlating the genetic distances of the different astrovirus sequences with their bat host genetic distances. We had originally expected that if the transmission route of astrovirus sequences resembles the pattern of host gene flow, genetic co-variation between astroviruses and host populations should be detected, especially across regions. The reason why no such correlation was found for *M. nattereri* and its associated astroviruses could be due to strong differences in population size (hence genetic drift) and mutation rate between bats and viruses. In contrast to higher Eukaryotes such as the bat host, RNA viruses are subject to higher selective pressures and combined with a high mutation rate allow continuous and rapid adaptation to changing environmental conditions [73, 76, 77]. Coupled with large population sizes, virus evolution can thus already be observed within very short time scales of weeks to months [74, 75]. The frequent fluctuations in the prevalence of viral populations (e.g. bottlenecks) and hence the higher genetic drift they face might prevent these populations from showing patterns of isolation-by-distance [78]. Moreover, our virus sampling could only be performed during the summer period since only some of the autumn swarming sites are known so far. Additionally, due to logistic reasons exact sampling dates differed between the sampling localities, which could blur the signal if some haplotypes or putative viral species are more abundant in different periods (see Additional file 1: Table S1). Finally, although large from a virology perspective, the number of samples with viral material was relatively limited to perform population genetics analyses. This combined with the variations in sampling times could confound our analysis if viruses show quick temporal variation in prevalence and/or turnover. A further possible explanation for the lack of a genetic correlation between hosts and viruses is that at swarming sites bats may not only interact in ways that lead to gene flow. Multiple mating and contact between individuals that does not result in successful mating might also represent transmission opportunities for viruses that are not reflected within the host genetic pattern.

## Conclusions

In summary, our findings suggest that for *M. nattereri* within Germany, swarming sites act as stepping-stones for gene flow, as indicated by an overall isolation-by-distance pattern and the absence of such a significant pattern on a local scale. The observed population genetic structure indicates that no apparent strong barriers to gene flow exist within our study area for the bat host. While putative viral species were mostly shared between geographic regions, no haplotypes were shared for any putative viral species. Despite the observed genetic differentiation between the three geographic regions in the bat host and to a certain extent also in the harbored astroviruses, we did not detect a correlation between host and virus genetic distances. This could potentially be due to differences in genetic drift, selective pressure, population size and mutation rate between bats and viruses. Further studies with a higher astrovirus sample size and with specific simultaneous sampling during autumn mating at swarming sites are required to shed further light on the host-virus relationship between bats and their astroviruses.

## Additional files

Additional file 1: Supplementary material. **Figure S1.** Summary of the log-likelihood values from the 20 independent runs conducted with Structure for the number of genetic clusters (*K)* set to a minimum of 1 and a maximum of 10. The left graph shows the log-likelihood results of the runs for each *K*, whereas the right graph shows Delta *K* plotted against *K*. The most likely number of genetic clusters is three using both methods. **Figure S2.** Comparisons of the STRUCTURE runs for K = 3 with (top) or without (bottom) the LOCPRIOR option. **Table S1.** Table of sampling times and associated screening for astroviruses. AstV = astrovirus, BY = Bavaria, MV = Mecklenburg Western Pomerania, NRW = North Rhine Westphalia. AstV positive samples with a length of less than 279 nt could not be assigned to a specific haplotype, but are nevertheless included in the number of AstV positive samples presented here. **Database S1.** R-script used for the permutation test. With this script we tested how likely it is to have no overlap in virus haplotypes across regions and whether virus haplotypes are more different between regions than expected by chance. (DOCX 318 kb)

Additional file 2: Genotype table *Myotis nattereri.* Table of alleles per locus for each individual bat used for the population genetic analyses on the Natterer’s bat (*Myotis nattereri*) determined using Genemapper v 5.0 (Applied Biosystems). (XLSX 75 kb)
