# Supplementary material for: Evidence for genetic variation in Natterer’s bats (Myotis nattereri) across three regions in Germany but no evidence for co-variation with their associated astroviruses
Source: BMC Evol Biol. 2017 Jan 5;17:5. doi: 10.1186/s12862-016-0856-0 (PMC5217449; doi:10.1186/s12862-016-0856-0)
Supplement: Supplementary file 1 — Supplementary material. Figure S1. Summary of the log-likelihood values from the 20 independent runs conducted with Structure for the number of genetic clusters (K) set to a minimum of 1 and a maximum of 10. The left graph shows the log-likelihood results of the runs for each K, whereas the right graph shows Delta K plotted against K. The most likely number of genetic clusters is three using both methods. Figure S2. Comparisons of the STRUCTURE runs for K = 3 with (top) or without (bottom) the LOCPRIOR option. Table S1. Table of sampling times and associated screening for astroviruses. AstV = astrovirus, BY = Bavaria, MV = Mecklenburg Western Pomerania, NRW = North Rhine Westphalia. AstV positive samples with a length of less than 279 nt could not be assigned to a specific haplotype, but are nevertheless included in the number of AstV positive samples presented here. Database S1. R-script used for the permutation test. With this script we tested how likely it is to have no overlap in virus haplotypes across regions and whether virus haplotypes are more different between regions than expected by chance. (DOCX 318 kb) [file 12862_2016_856_MOESM1_ESM.docx]

**Supplementary material**

**
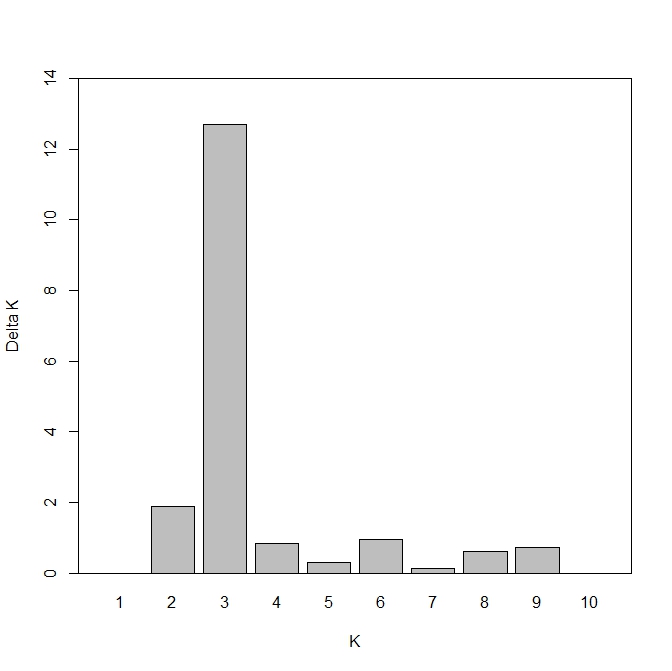

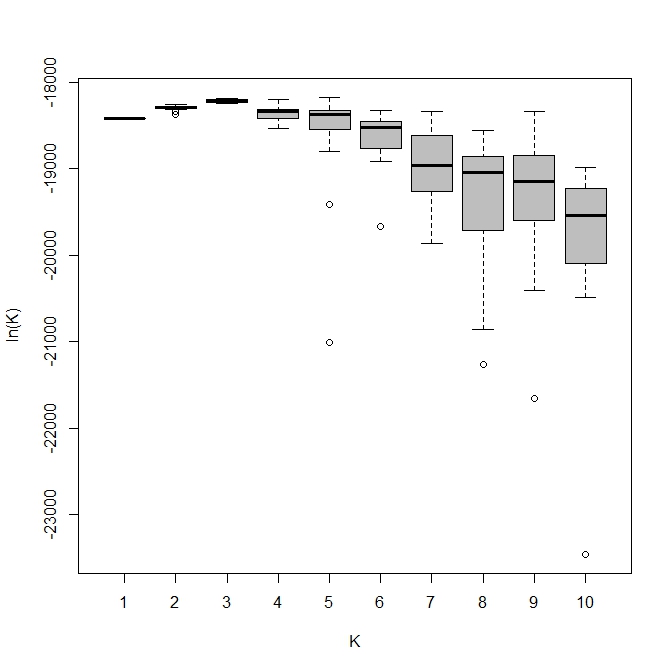
**

**Figure S1** Summary of the log-likelihood values from the twenty independent runs conducted with Structure for the number of genetic clusters (*K)* set to a minimum of 1 and a maximum of 10. The left graph shows the log-likelihood results of the runs for each *K*, whereas the right graph shows Delta *K* plotted against *K*. The most likely number of genetic clusters is three using both methods.


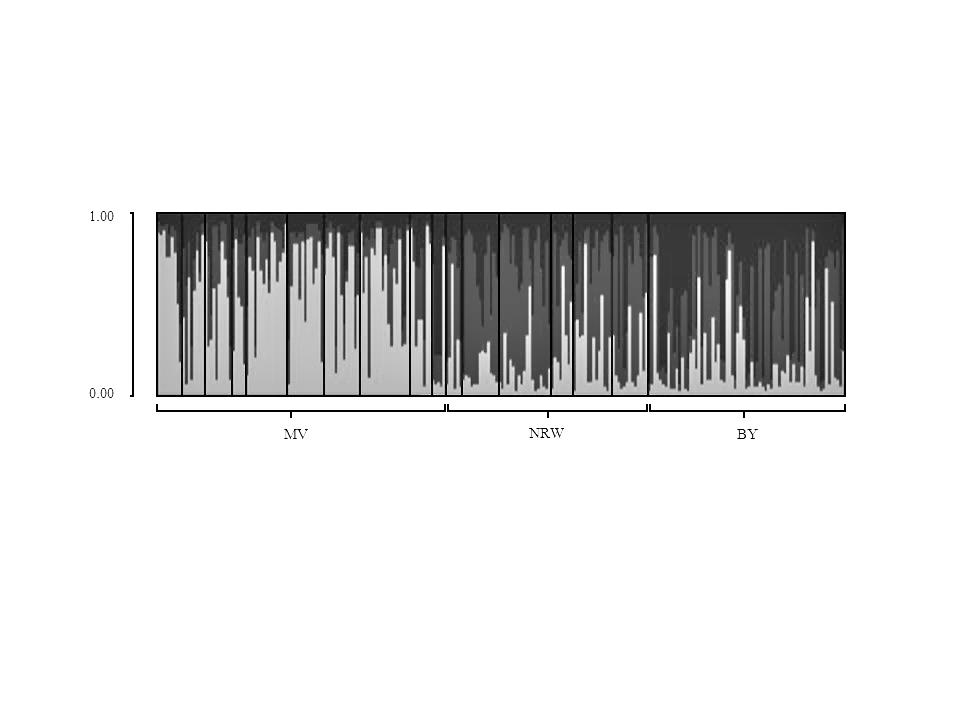

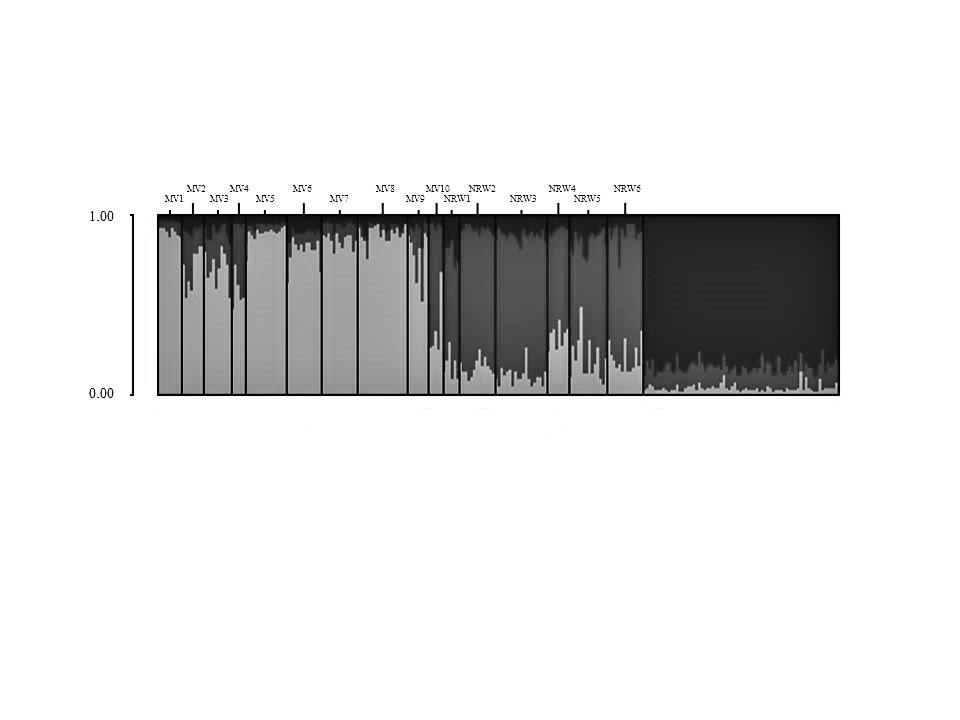

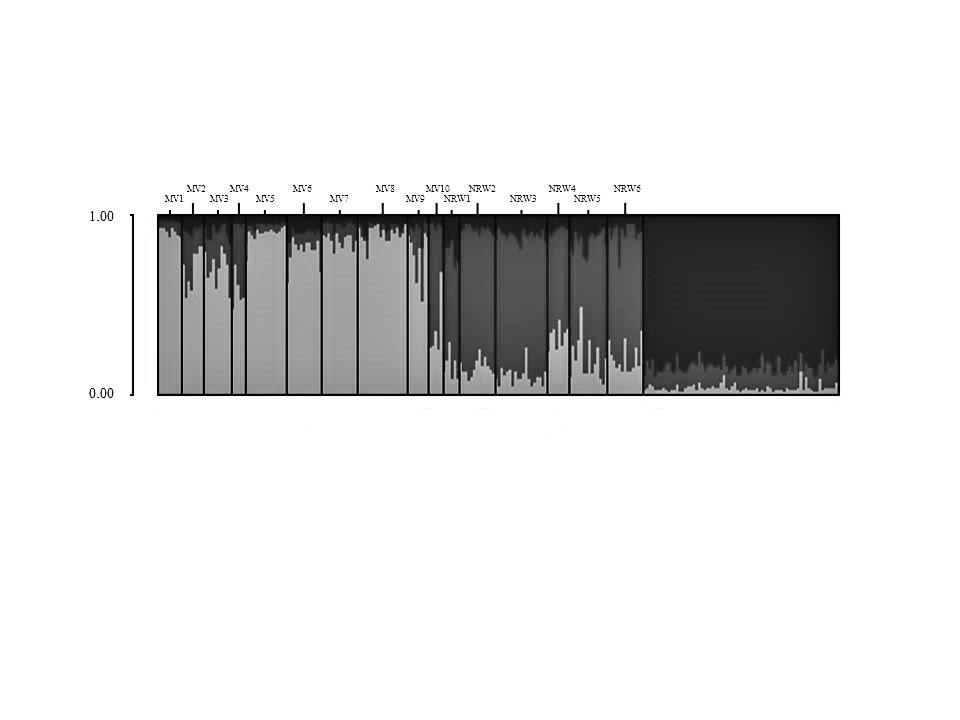


**Figure S2** Comparisons of the Structure runs for *K*=3 with (top) or without (bottom) the Locprior option.

**Table S1** Table of sampling times and associated screening for astroviruses.

| **State** | **sampling date** | **individuals sampled** | **AstV positive samples** | | **AstV samples assigned to a specific sequence type** |
| --- | --- | --- | --- | --- | --- |
| **BY** | 18.05.2011 | 38 | 23 | 17 | |
|  | 07.-09.08.2012 | 12 | 5 | 5 | |
|  | 18.05.2013 | 13 | 5 | 4 | |
|  | 07.08.2013 | 33 | 17 | 10 | |
|  | 08.05.2014 | 27 | 2 | 2 | |
|  | 13.05.2014 | 30 | 3 | 3 | |
|  | 18.-19.05.2014 | 24 | 1 | 1 | |
| **MV** | 23.-25.07.2012 | 6 | 2 | 1 | |
|  | 23.07.2013 | 3 | 0 | 0 | |
|  | 21.08.2013 | 10 | 7 | 4 | |
| **NRW** | 26.-27.07.2014 | 37 | 18 | 14 | |
|  | 06.-07.08.2014 | 16 | 11 | 6 | |
|  | 13.08.2014 | 6 | 1 | 1 | |
|  | 22.08.2014 | 4 | 3 | 2 | |
|  | 23.-24.09.2014 | 6 | 5 | 2 | |
|  | 01.10.2014 | 5 | 3 | 1 | |

AstV = astrovirus, BY = Bavaria, MV = Mecklenburg Western Pomerania, NRW = North Rhine Westphalia. AstV positive samples with a length of less than 279 nt could not be assigned to a specific sequence type, but are nevertheless included in the number of AstV positive samples presented here.

**Database S1** R-script used for the permutation test. With this script we tested how likely it is to have no overlap in virus sequence across regions and whether virus sequences are more different between regions than expected by chance.

#Sets the path, reads the file

setwd("Path-to-the-folder-with-the-file ")

dat<-read.csv("Input-csv-file.csv",h=TRUE)

#How many permutations to run

Nperm<-100000

#Simulations (in fact permutations)

#Objects to store the results

perm<-as.data.frame(matrix(0,length(dat[,1]),Nperm))

Overlap<-vector()

#Loop to run the permutations

for (i in 1:Nperm) {

perm[,i]<-sample(dat[,1],replace=FALSE) #Permutations

tab<-table(perm[,i],dat[,2]) #Cross-table to figure out overlaps

tabSum<-apply(tab/tab,1,sum) #Calculates overlaps between regions

Overlap[i]<-length(which(tabSum>0)) #Counts overlaps between regions and

stores them

}

#Plots an histogram of overlap

hist(Overlap,breaks=c(seq(-0.5,10,by=1)))

#Calculates the probability that by chance alone, no overlap in viral sequence would exist between regions

length(which(Overlap== 0))/Nperm
